# Human Health Risks Associated with the Consumption of Wild Foods and Geophagic Material Along the Great Dyke and Surrounding Areas in Zimbabwe

**DOI:** 10.3390/ijerph23080957

**Published:** 2026-07-24

**Authors:** Aaron Chigona, Farai Mapanda, Chakare Benhura, Justice Nyamangara, Phillip Nyamugafata, Menas Wuta, Ntandokamlimu Nondo, Sessely Mavunga, Takudzwa Nota

**Affiliations:** 1 Department of Agricultural and Biosystems Engineering, University of Zimbabwe, 1 Mt Pleasant Drive, Harare P.O. Box MP 167, Zimbabwe; 2 Environmental Management Agency, 685/6 Lorraine Drive/Faber Road, Harare P.O. Box CY 385, Zimbabwe; ntando.nondo@ema.co.zw (N.N.); sessely.mavunga@ema.co.zw (S.M.); takudzwa.nota@ema.co.zw (T.N.); 3 Department of Soil Science and Environment, University of Zimbabwe, 1 Mt Pleasant Drive, Harare P.O. Box MP 167, Zimbabwe; faraimaps@yahoo.com (F.M.); pnyamugafata@gmail.com (P.N.); wokwawuta@gmail.com (M.W.); 4 Department of Nutrition, Dietetics and Food Sciences, 1 Mt Pleasant Drive, Harare P.O. Box MP 167, Zimbabwe; cbenhura@science.uz.ac.zw; 5 Climate Change and Food Security Institute, Marondera University of Agricultural Sciences and Technology, Plot 15 Longlands Road, Marondera P.O. Box 35, Zimbabwe; jnyamangara@muast.ac.zw

**Keywords:** cancer risk, hazard index, hazard quotient, health risk assessment, serpentinic ultramafic geology, toxic geogenic contaminants, wild foods

## Abstract

**Highlights:**

**Public health relevance—How does this work relate to a public health issue?**

- Wild foods and geophagic materials from serpentinic ultramafic (SUG) and surrounding granitic (GG) geological environments contain toxic geogenic contaminants (TGCs) exceeding WHO permissible limits.
- Consumption of TGC-contaminated wild foods exposes communities to carcinogenic and non-carcinogenic health risks.

**Public health significance—Why is this work of significance to public health?**

- Fifty percent (50%) of the wild foods studied exceeded WHO/FAO tolerable health risk levels.
- *Ficus sycomorus* and geophagic materials sourced from the Great Dyke (SUG environment) presented the highest carcinogenic and non-carcinogenic risks to consuming communities.

**Public health implications—What are the key implications or messages for practitioners, policy makers and/or researchers in public health?**

- Medicinal plants taken as infusions significantly reduce TGC intake and associated health risks.
- Environmental health policies must increase awareness among communities on the risks associated with consuming wild foods and geophagic materials from the Great Dyke and surrounding areas.

**Abstract:**

**Background:** Wild foods and geophagic material are important in the nutrition of communities along Zimbabwe’s Great Dyke, a serpentinic ultramafic (SUG) and surrounding granitic geological (GG) environment. However, the health risks associated with consumption of these in terms of toxic geogenic contaminants (TGC) is largely unknown. The study objective was to evaluate the health risks associated with consuming wild foods and geophagic materials from these environments. **Methods:** Health risk assessments were determined using the hazard quotient, hazard index (HI) and life cancer risk (LCR) for 10 TGCs in wild foods and geophagic material. **Results:** High human health risks (HI ≥ 1) were found in six wild foods and geophagic material under the SUG environment compared with five wild foods and geophagic material under the GG environment. Wild foods and geophagic materials exceeded the tolerable risk level of 1 × 10^−4^. Their LCR values ranged from 2.11 × 10^−1^ to 6.83 × 10^−7^. All TGCs had high LCR values above the tolerable risk level, except for Cu, Fe, Mn and Zn. **Conclusions:** Infusion reduced the LCR values for TGCs, with only Pb in the GG environment decreasing to below tolerable risk level. Communities should apply selective consumption and avoid *F. sycomorus* and geophagic materials with cancer risks.

## 1. Introduction

Globally, toxic geogenic contaminants’ (TGCs’) accumulation in wild foods, medicinal plants and geophagic material poses public health threats, with widespread contamination documented across mineral-rich regions such as Asia and Latin America [1]. Within the African continent, this issue is mirrored in several mineralized and mining landscapes; for instance, high levels of geogenic and anthropogenic TGCs in wild foods, leafy vegetables, and geophagic clays have been documented in regions like the Witwatersrand basin in South Africa, the Copperbelt of Zambia, and various artisanal gold mining communities in Nigeria, Ghana, and East Africa [2]. The consumption of wild foods (fruits, medicinal plants, insects and rodents) and geophagic materials has long been an important component of rural communities’ sustenance, especially during periods of food scarcity, providing essential nutrients and serving as dietary supplements. Despite their nutritional value, these wild foods can also accumulate harmful TGCs such as heavy metals, particularly when gathered from environments naturally enriched with geogenic and anthropogenic contamination from mining and farming [3]. Long-term exposure to such TGCs can lead to severe health issues that include cancer, organ failure and neurological disorders [4]. The lifetime consumption of TGC-contaminated wild foods has not been reported with respect to adverse human health risks in the Great Dyke and related geological environments in Zimbabwe. Such studies are critical to the livelihoods of communities who depend on wild foods as a coping strategy in the advent of climate change.

Studies have shown that plants growing in soils with high concentrations of TGCs can bioaccumulate these contaminants [5,6]. In Zimbabwe’s Great Dyke, a serpentinic ultramafic geological (SUG) environment, significant amounts of TGCs that include chromium (Cr), nickel (Ni), lead (Pb), arsenic (As), cadmium (Cd), and zinc (Zn) have been found to be in high concentrations in soils [7]. These TGCs have potential to contaminate wild foods (fruits, medicinal plant, insects, rodents) and geophagic materials harvested from these SUG environments. Communities consuming TGC-contaminated wild foods have been observed to exhibit poisoning symptoms and signs of chronic diseases such as Itai-Itai disease (from Cd), gastrointestinal ulceration (from Cr) and dermatitis (from Ni) [8,9,10,11], particularly among the elderly, children, and pregnant women, who are at increased risk due to their vulnerability to the adverse effects of these contaminants [12]. However, the human health risks associated with the consumption of wild foods from such environments are largely unknown. Assessment of the human health risk would be a critical step in finding interventions in reducing risks from both policy and behavioral perspectives.

Human health risk assessment methods for contaminated wild foods follow a multi-step process of hazard identification, exposure assessment, toxicity assessment, and risk characterization using estimated daily intake (EDI), chronic daily intake (CDI) and indices such as the hazard quotient (HQ), hazard index (HI), and lifetime cancer risk (LCR) [13]. These methods are recommended by researchers because they account for local dietary ingestion rates, cultural practices, and specific exposure pathways [14].

The objective of this study was to determine the human health risks associated with the selected wild foods and geophagic material consumed by communities along the SUG and in the surrounding granitic geological (GG) environments in Zimbabwe. The outputs are expected to provide a foundation for environmental policy and sustainable community-level interventions to reduce TGC dietary exposure and safeguard public health in SUG and GG environments that are geogenically and anthropogenically contaminated.

## 2. Materials and Methods

### 2.1. Description of Study Site and Site Selection

**Figure 1:**
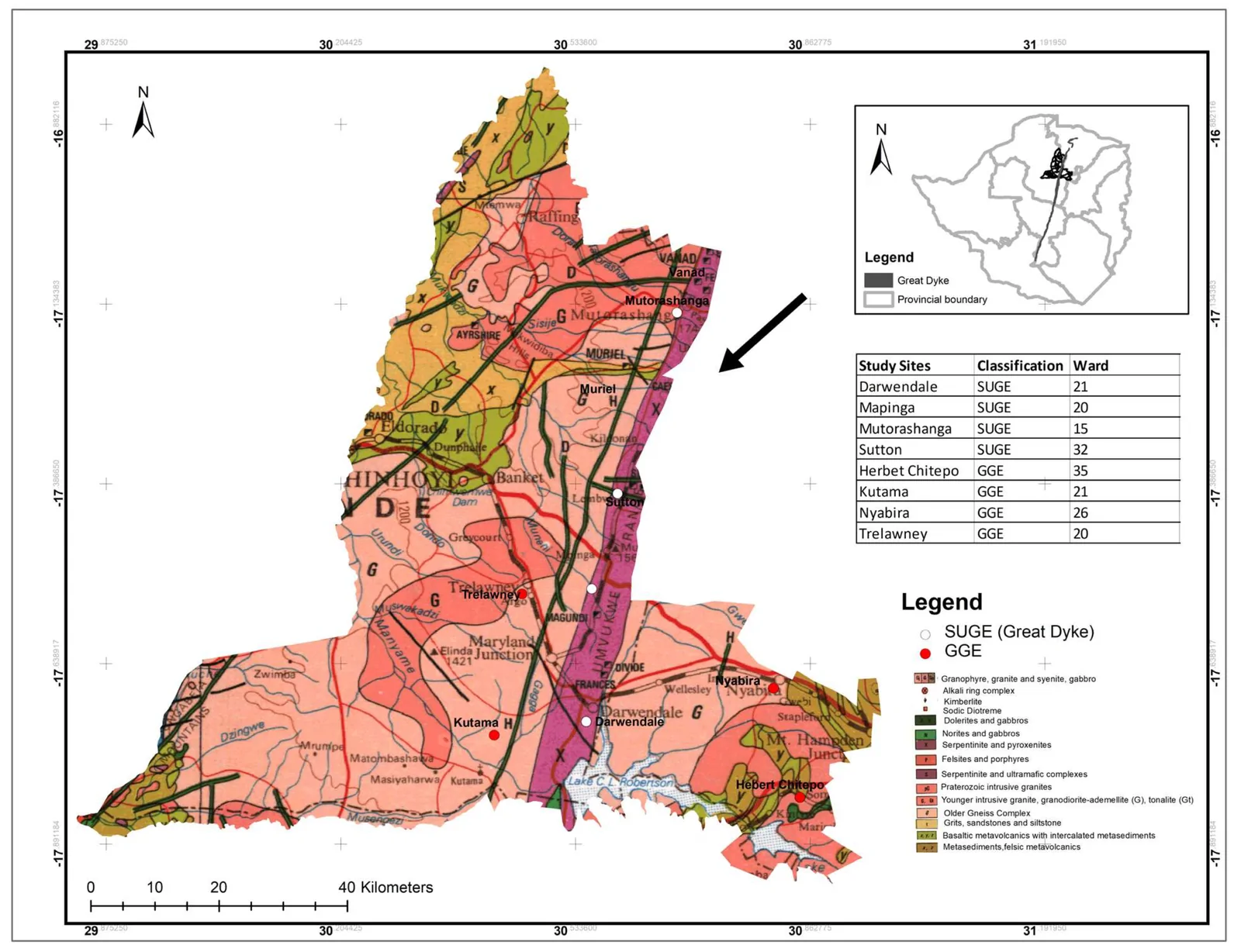
Study site and sampling locations along the Great Dyke and surrounding areas in Zimbabwe.

The Great Dyke is a mountainous landform formed by the intrusion of mafic and ultramafic rocks, extending from northeast to southwest Zimbabwe and covering approximately 291,722 ha (0.8% of the country’s total area). The study area covers 6 wards (15, 20, 21, 26, 32 and 35) with an area of about 123,000 ha (Figure 1), of which about 40% is in the Great Dyke. The six wards have a total population of 149,133 [15]. The dominant land uses are mining (illegal, small-scale, and large-scale) and cropping (subsistence and commercial), while the remaining areas are mostly woodlands and grasslands in which the wild foods are found. These wild foods include medicinal plants, mushrooms, fruits, honey, rodents, and edible insects.

The landform along the Great Dyke (study site) provides a unique geological environment, with nickel and chromium mineral deposits that are potential geogenic contaminants. The study area lies within Agroecological Zone IIa, with an average maximum temperature of 25.5 °C and a mean annual rainfall of 850 mm [16]. The predominant vegetation in this region is miombo woodland. Major tree species include *Julbernardia* spp. and *Brachystegia* spp. [17]. The soils of the SUG environment vary along the dyke and differ from those of the surrounding granitic geological environment due to distinct rock formations and weathering processes [7]. Soil types along the Great Dyke (SUG environment) are medium to heavy textured, while those in the GG environment are light textured [18].

The selection of study sites was adopted from a study by Chigona et al. (2026) that focused on wild foods consumed along the Great Dyke and surrounding environments, including their TGC concentrations [19]. The sites were Darwendale, Mapinga, Mutorashanga and Sutton for the SUG environment and Herbert Chitepo, Kutama, Nyabira and Trelawney for the GG environment (Figure 1). On these sites, the primary focus of the study was to establish the human health risk associated with long-term consumption of the wild foods.

### 2.2. Estimation of Availability and Consumption Frequency of Wild Foods

Thirteen (13) wild foods, comprising 6 fruits, 5 medicinal plants, termites, rodents and geophagic material found in the two geological environments were selected for the study. The availability of selected wild foods throughout the year and the parts consumed were determined through household questionnaire surveys (n = 169) and focus group discussions with participants from the two geological environments [19]. The quantity and frequency of wild foods consumed were determined using ten (10) key informants from the two geological environments. The 10 selected key informants were drawn from participants who had stayed in the study area for at least 10 years [19]. The key informants also provided information on wild food processing and ingestion methods, as well as the months and days when the wild foods were consumed.

Key informants were asked to report their weekly consumption frequency for each wild food item, expressed as the number of consumption events per week. Regarding medicinal plant use, key informants freely provided information on the preparation of medicinal plants (*Erythrina abyssinica*, *Azanza garckeana*, *Asparagus laricinus*, *Amaranthus thunbergii*, and *Lippia javanica*), as well as on dosage and duration of consumption. The key informants were reminded of their rights to non-disclosure of information on medicinal plants’ dosage in line with the provisions of Ref. [20], Zimbabwe SI 61 of 2009, access to genetic resources and indigenous genetic resource-based knowledge regulation.

### 2.3. Estimation of Wild Fruit Portions Consumed

Key informants provided the number of each wild fruit consumed per meal. To convert the number of fruits consumed to mass consumed per meal, the edible portions of six wild fruits, *Strychnos cocculoides*, *Azanza garckeana*, *Euclea natalensis*, *Ziziphus mucronata*, *Ficus sycomorus*, and *Flacourtia indica*, were quantified in the laboratory. Ten (10) randomly selected fruits per species were cleaned using Type 2 purified water. The non-edible parts (seeds, shells) were removed using a scalpel and tweezers. The fresh edible pulp masses of each fruit species were weighed individually on a calibrated analytical balance (model: Sartorius Practicum, Göttingen, Germany), and the total was averaged. The meal portions were estimated from the average mass of each fruit species and the number of fruits consumed per meal. Wild fruit intake per week was estimated from meal portions and weekly consumption frequency. From these figures, the daily intake (DI) or ingestion rates (IR) were computed for each wild fruit species (Equation (1)):

(1) $$IR=\frac{\left(FC\;PS\right)}{T}$$

where:

*IR* = ingestion rate (g/day);

*FC* = frequency of consumption (#);

*PS* = amount consumed per eating occasion (g);

*T* = duration over which the frequency is measured (days).

### 2.4. Estimation of Medicinal Plants’ Consumption Dosages

Key informants indirectly provided the dosage of medicinal plants administered by demonstrating the size and number of barks (*Erythrina abyssinica*), roots (*Asparagus laricinus*), and leaves (*Azanza garckeana*, *Amaranthus thunbergii*, *Lippia javanica*), as well as the volume of water to which these parts were added. The demonstration samples were placed in khaki envelopes, labelled, and taken to the laboratory for weighing, since there were no weighing facilities in the field.

To convert these demonstrations of parts and numbers (portions) into dosages, the following approaches were used. For the selected medicinal plants, 10 portions of each plant part, provided by key informants, were weighed on a calibrated analytical balance and averaged. The fresh weight was converted to dry weight by oven drying at 60 °C for 120 h. Oven-dried samples were ground, sieved through a 2 mm sieve, and stored in khaki envelopes before infusion preparation with type 2 purified water.

The infusions of the medicinal plants were prepared by adding the mean weight of ground and sieved medicinal plant parts to 300 mL of boiled water, which was allowed to cool to room temperature. For *Erythrina abyssinica*, the infusion was prepared in the laboratory by adding 7.14 g of ground, sieved sample to 300 mL of boiled water and allowing it to cool to room temperature. For *Asparagus laricinus*, the infusion was prepared by adding 8.40 g of ground, sieved sample to 300 mL of boiled water and then leaving it to cool to room temperature. For *Azanza garckeana*, the infusion was prepared by adding 6.57 g of ground, sieved sample to 300 mL of boiled water and leaving it to cool to room temperature. For *Amaranthus thunbergii*, the infusion was prepared by adding 5.89 g of ground, sieved sample to 300 mL of boiled water and leaving it to cool to room temperature. For *Lippia javanica*, the infusion was prepared by adding 3.69 g of ground, sieved sample to 300 mL of boiled water and then leaving it cool to room temperature. The resultant supernatant represented the ingestion rate for the five (5) medicinal plants (Equation (1)).

### 2.5. Estimation of Termite and Rodent Portions Consumed

Key informants provided the quantities of termites and rodents consumed per meal using cup demonstrations and numbers, respectively. The conversion of the cup of termites and rodents consumed per meal to mass was performed in the laboratory. Termites collected from the field were screened by removing debris and other foreign material. After screening, the mass of ten (10) replicate cups filled to the brim with termites was determined and averaged. Ten (10) replicate individual termites were selected randomly from each of the ten cups and weighed. The resultant weights were averaged to determine the average mass of an individual termite. This average termite mass was used to determine the average number of termites in a cup.

Ten (10) replicate traditionally trapped and dried rodents, with internal organs and fur removed, were weighed on a calibrated analytical balance (model: Sartorius Practicum, Germany) and averaged. Weekly rodent meal portions were estimated from the average mass of each rodent and the number consumed per week. The rodent weekly intake was then used to compute the daily intake rate (Equation (1)).

### 2.6. Estimation of Geophagic Material Consumed per Day

Key informants provided data on the number of handfuls of geophagic material (termite-tree-wrap soil) consumed per week. To convert this number to mass, ten (10) replicate handfuls of geophagic material from different key informants were placed in labelled plastic bags and transported to the laboratory. The samples were oven-dried at 60 °C until constant weight was achieved. The sample was then weighed on a calibrated analytical balance (model: Sartorius Practicum, Germany), and the mean was calculated. The weekly geophagic material intake was then used to compute the daily intake rate (Equation (1)).

### 2.7. Determination of TGC in Wild Foods Consumed

Determinations of total TGCs (As, Cd, Co, Cr, Cu, Fe, Mn, Ni, Pb and Zn) in wild fruits (n = 181), medicinal plants (n = 138), termites (n = 30), rodents (n = 63), and geophagic material (n = 72) were reported by Chigona et al. (2026) [19]. In addition, TGCs (Cd, Co, Cr, Cu, Fe, Mn, Ni, Pb and Zn) in infusions of medicinal plants (*Erythrina abyssinica*, *Asparagus laricinus*, *Azanza garckeana*, *Amaranthus thunbergii* and *Lippia javanica*) were determined in the supernatant, which was analyzed by flame atomic absorption spectrophotometry (FAAS) (GBC Savant AA Spectrophotometer, GBC Australia) [21]. The concentrations of TGCs (mg/L) in the infusions were then expressed as the mass of TGCs (mg) per kg of the medicinal plant.

### 2.8. Data Analysis

Data were subjected to descriptive analysis covering the frequency of consumption, ingestion rate, and hazard indices. Standard errors for these parameters were computed using the error-propagation method [22]. Human health risks were computed using hazard quotients, hazard indices, and lifetime cancer risks for the SUG and GG environments using TGC concentrations in Table 1. R Studio (4.3.1 version 2025) was used for graphical data visualization.

#### 2.8.1. Estimation of Daily TGC Intake from Wild Foods Consumption

Estimated daily intake (EDI) and chronic daily intake (CDI) were calculated using Equation (2) [23] for carcinogenic and non-carcinogenic effects. For EDI, the actual average time a person stayed exposed to contamination is used. In the case of CDI, the average life expectancy period is used.

(2) $$EDI=\frac{C\times IR\times EF\times ED}{BW\times AT}$$

here:

*EDI* = estimated daily intake (mg/kg body weight/ day);

*C* = concentration of the TGC in the wild food type (mg/kg);

*IR* = ingestion rate (kg/day);

*EF* = exposure frequency (days/year);

*ED* = exposure duration (years);

*BW* = average body weight of the consumer (kg);

*AT* = averaging time (days); for non-carcinogenic effects, *AT* = *ED* (42) × 365 days, for carcinogenic effects, AT = ED (70) × 365 days;

*CDI* = chronic daily intake (mg/kg body weight/day) for carcinogenic effect AT = ED (70) × 365 days.

The average body weight of adults in the SUG and GG communities was computed at 65 kg based on epidemiological data collected from the eight health centers in the study area and verified against general population data [15].

#### 2.8.2. Hazard Quotient Derivation

The hazard quotient (HQ) was calculated using the global standard oral reference doses (R*f*D) for the TGCs, obtained from the USEPA Integrated Risk Information System (IRIS) (Table 2), and the EDI. The hazard quotient (HQ) was then computed for each TGC in each wild food using Equation (3) [23]:

(3) $$HQ=\frac{EDI}{RfD}$$

where:

*EDI* = estimated daily intake of TGC (mg/kg/day);

R*f*D = standard oral reference doses for TGC (mg/kg/day).

**Table 2:** USEPA IRIS standard oral reference doses (R*f*D) for heavy metals TGCs).

*TGC*	R*f*D (mg/kg Body Weight/Day)
As	0.00006
Cd	0.001
Co	0.03
Cr (VI)	0.003
Cu	0.04
Fe	0.30
Mn	0.14
Ni	0.02
Pb	0.0035
Zn	0.30

#### 2.8.3. Hazard Index Derivation

The hazard index (HI) for individual TGCs in wild foods, individual wild foods, and the wild food basket consumed in the SUG and GG environments was calculated using Equation (4) [23]. This approach allows for the cumulative assessment of multiple TGCs, providing a more comprehensive evaluation of potential health risks associated with the consumption of wild foods in the study area. Interpretation of the HI value was as follows:

HI < 1 indicates that the cumulative exposure to the contaminants is unlikely to cause adverse health effects.

HI ≥ 1 suggests a potential for non-carcinogenic health risks and warrants further attention.

(4) $$HI=\sum_{i=1}^{n}HQi$$

where:

*HQi* represents the hazard quotient of the *i*th contaminant;

*n* is the total number of TGCs considered in the assessment.

#### 2.8.4. Lifetime Cancer Risk

Lifetime cancer risk (LCR) was calculated using Equation (5) [24]. LCR value of 1 × 10^−6^ means 1 in a million chance of developing cancer over a lifetime, and 1 × 10^−4^ means 1 in 10,000 chances (often considered an upper acceptable risk) [25].

(5) LCR=CDI×CSF

where:

*CDI* = chronic daily intake of TGC (mg/kg/day);

*CSF* = cancer slope factor (oral) (mg/kg/day).

### 2.9. Methodology Summary

**Figure 2:**
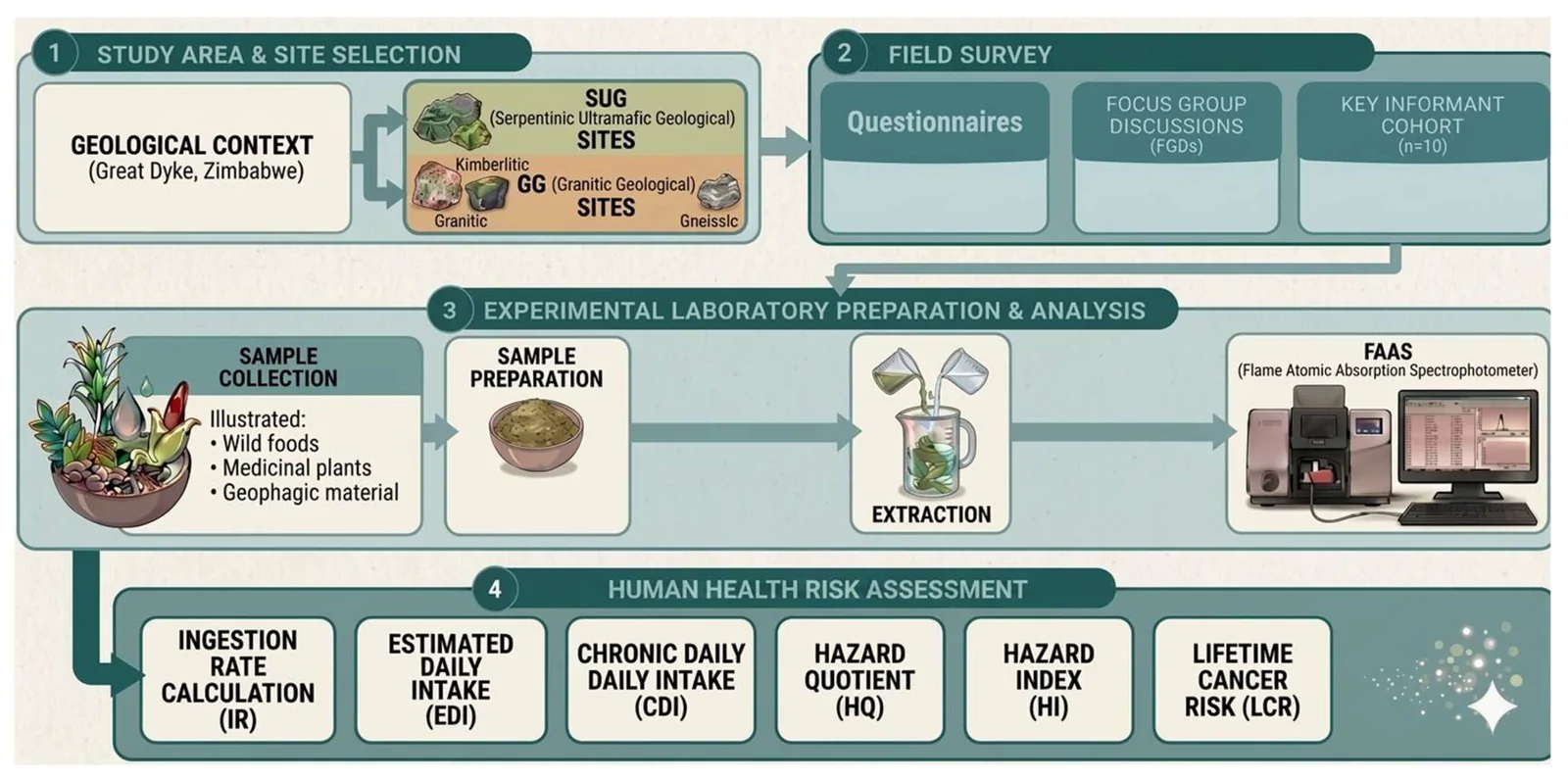
Graphical summary of the methodology.

Figure 2 shows the process flow of the methods and approaches used in the study.

## 3. Results

### 3.1. TGC Concentration in Medicinal Plant Infusions from the SUG and GG Environments

TGC in medicinal plants recorded significant differences in 80% of the elements analyzed, with at least two to four elements showing the differences, as shown in Table 3. Five medicinal plants (*A. garckeana*, *E. abyssinica*, *A. laricinus*, *A. thunbergii*, and *L. javanica*) recorded 1 to 5 TGC, indicating significant differences between the two geological environments. Copper (Cu), Fe, Mn, Pb, and Zn concentrations were not significantly different (*p* > 0.05) in the two geological environments. Cadmium and Cr were not detected in all the medicinal plant infusions, in contrast to observation of the same, while cobalt (Co) and Ni were not detected in at least one of the five medicinal plants (Table 1 and Table 3). Following infusions, all TGCs concentrations went below the permissible limits [26] except for Cu, Ni and Pb.

### 3.2. Wild Food Daily Ingestion Rate

Wild food ingestion rates for fruits, termites, rodents, geophagic material and medicinal plants are presented in Table 4 and Table 5. Results showed that of the six fruits, *Ficus sycomorus* and *Strychnos cocculoides* had the highest ingestion rate (>50 g/day) and the longest availability period. *Macrotermes falciger* had the shortest availability window and the lowest ingestion rate. In contrast, *Ziziphus mucronate*, although it is among the fruits with the highest availability window, was the least preferred, with an ingestion rate of <4 g/day. The results show that animal-source protein (*Mastomys natalensis*) had a higher ingestion rate than insect-source protein (*Macrotermes falciger*). The geophagic material ingestion rate was among the top five most highly consumed wild food and dietary supplement.

For medicinal plants, the ingestion rates, in descending order, were *A. laricinus > E. abyssinica > A. garckeana > L. javanica* and *A. thunbergii*. Two medicinal plants were consumed as a powder and an infusion (*E. abyssinica* and *A. laricinus*), with *A. laricinus* consumed at a higher rate than *E. abyssinica.*

### 3.3. Human Health Risks Indices for Wild Foods from the SUG and GG Environments

**Figure 3:**
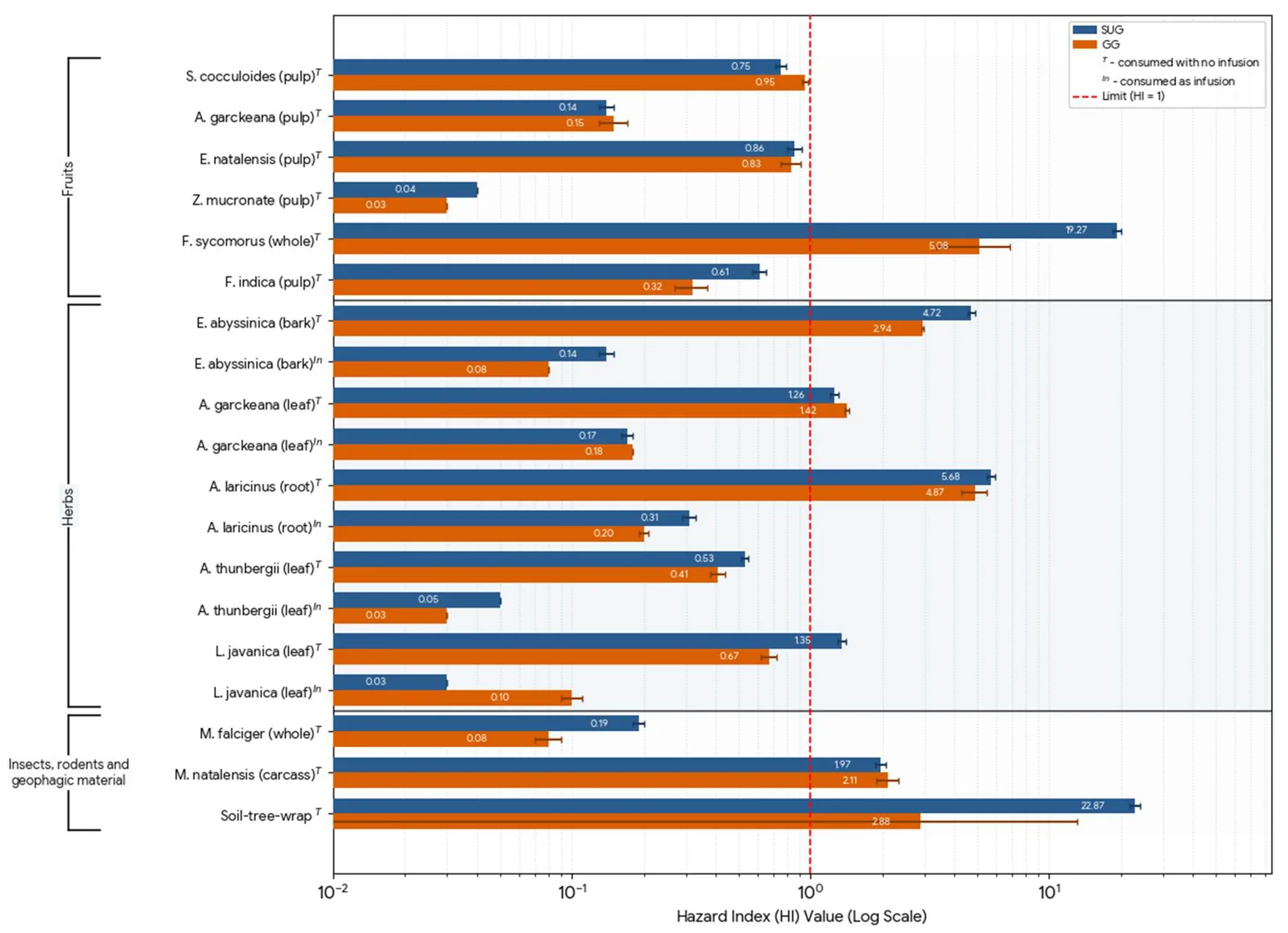
Hazard index (HI) from 10 TGCs for selected wild foods and geophagic material under the SUG and GG environment. Numbers on bars are means, and error bars denote standard error of mean. Letter “T” denotes HI for wild foods consumed with no infusion, and “In” denotes HI for wild foods consumed as infusion.

**Figure 4:**
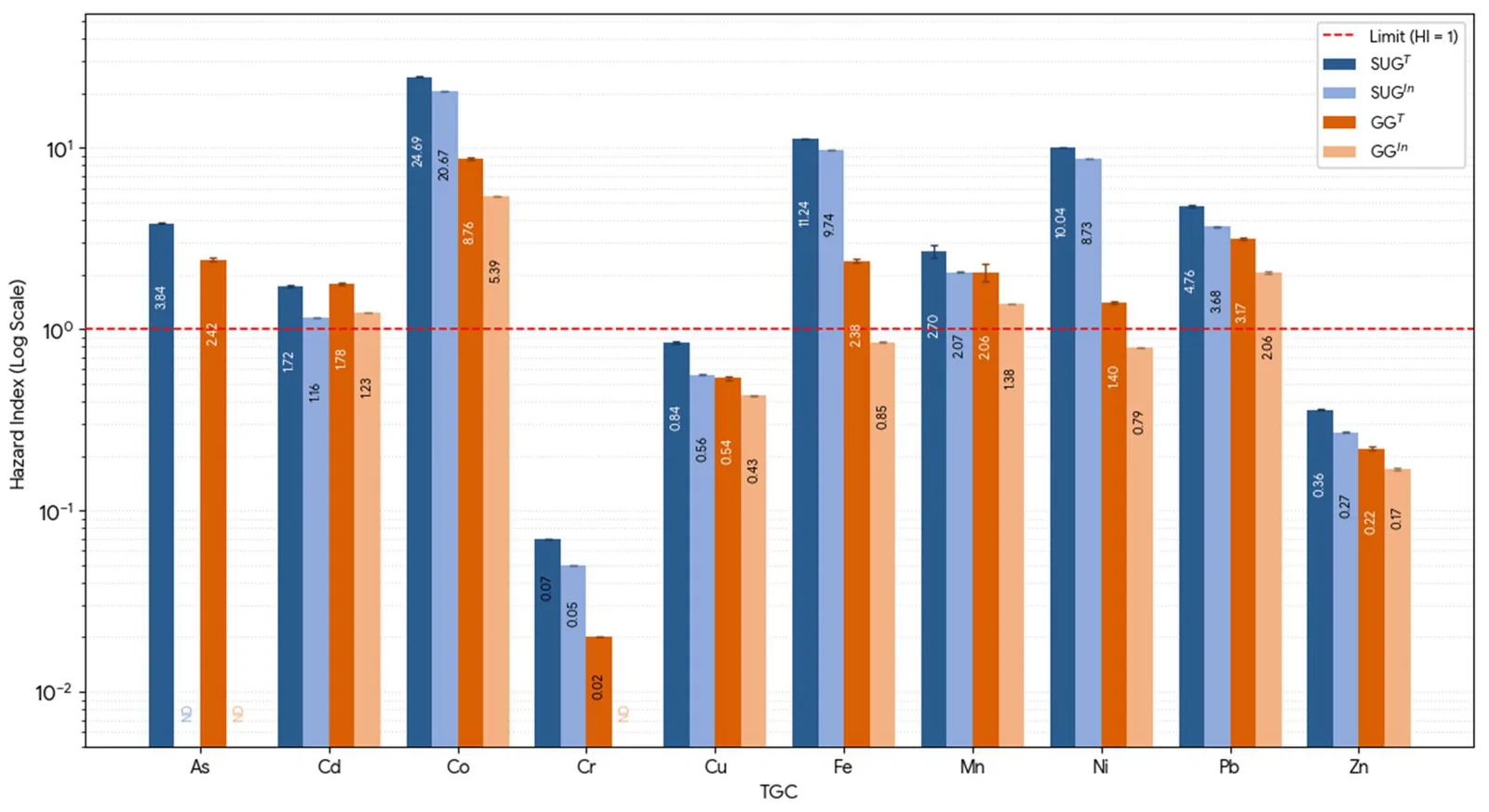
Hazard index (HI) for TGCs in 12 selected wild foods and geophagic material under the SUG and GG environments. Letter “(HI) T” denotes hazard index for wild foods plus medicinal plants consumed with no infusion; “(HI) In” denotes wild foods consumed with medicinal plants as infusion; NA denotes HI not assessed due to methodological challenges in determination of As in infusions. ND means not detected.

The results for human health indices’ HI were obtained for TGCs in (1) individual wild foods and geophagic material (Figure 3) and in (2) individual TGCs across the wild foods and geophagic material (Figure 4) in the SUG and GG environments. High human health risks (HI ≥ 1) were found in six wild foods (*A. garckeana*, *A. laricinus*, *F. sycomorus*, *E. abyssinica*, *M. natalensis* and *L. javanica*) and termite tree wrap (geophagic material) under the SUG environment compared with five wild foods (*A. garckeana*, *A. laricinus*, *F. sycomorus*, *E. abyssinica* and *M. natalensis*) and geophagic material under the GG environment (Figure 3). However, for (*A. garckeana*, *A. laricinus*, *E. abyssinica* and *L. javanica)* when consumed as infusions, the human health risk is significantly reduced (HI < 1). For *A. garckeana*, *E. abyssinica*, *L. javanica* and *M. natalensis* consumed whole (total, T), the driver for high HI was cobalt under both SUG and GG environments.

Cobalt was detected in all wild foods, with high human health risk under both geological environments, except for *E. natalensis* under the GG environment. The HI for TGCs in wild foods from the SUG and GG environments indicated that five wild foods (*S. cocculoides*, *A. garckeana*, *F. indica*, *A. thunbergii* and *M. falciger*) were safe for human consumption, with non-carcinogenic risk values below 1 (HI < 1).

TGC with a high human health risk under the GG environment accounted for 70% of the analyzed elements, whilst the remaining 30% (Cr, Cu, and Zn) had low HI (HI < 1). Arsenic was not detected in any of the five medicinal plants consumed as infusions. A total of 50% of the TGC in wild foods consumed in the GG environment, as an infusion, recorded a high HI (HQ ≥ 1), and 40% of them had a low HI (HI < 1), with three of them being like those under the SUG environment, except for Fe.

### 3.4. Wild Food Toxic Geogenic Contaminant Lifetime Cancer Risk for SUG and GG Environments

**Figure 5:**
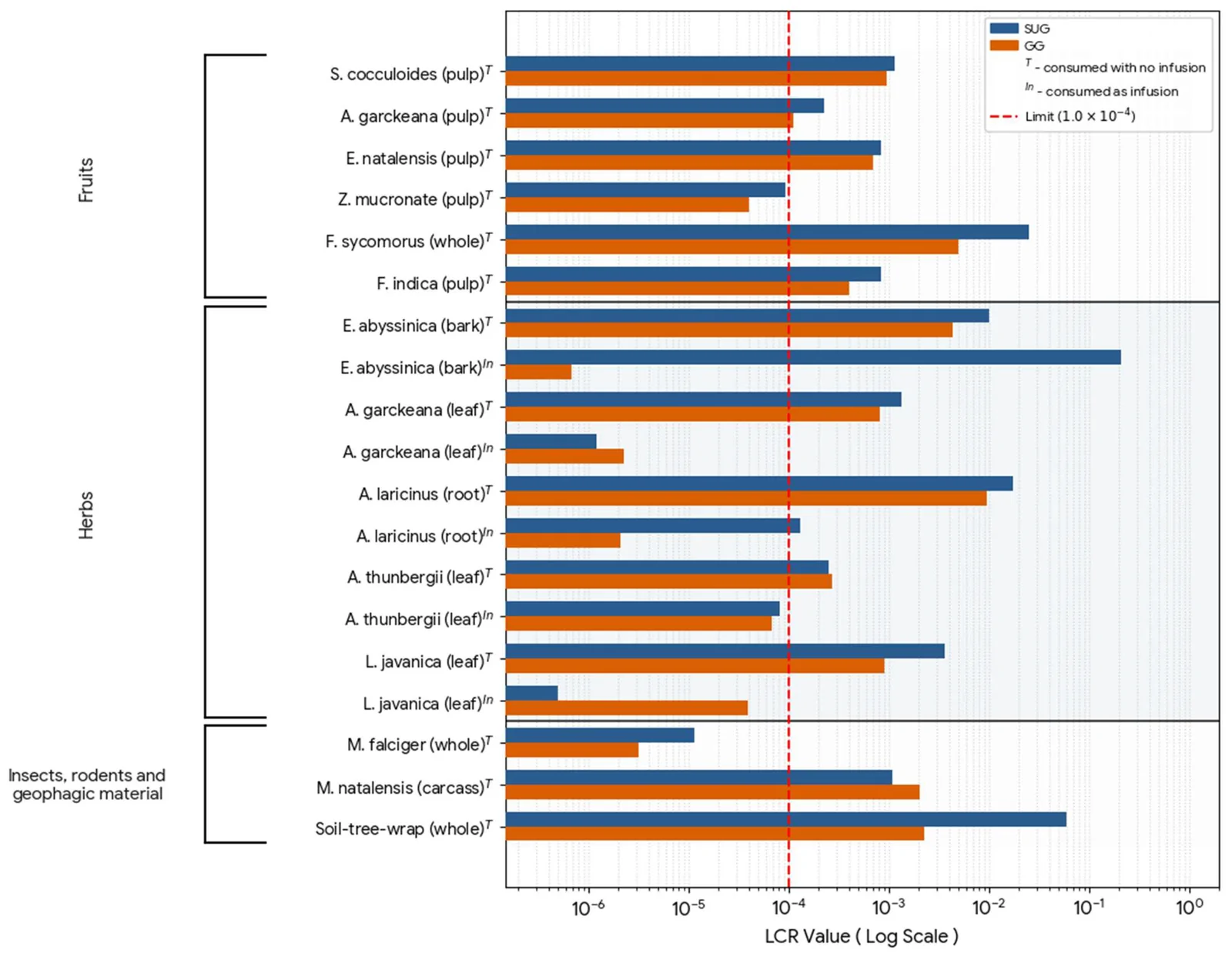
Lifetime cancer risk (LCR) for wild fruits, medicinal plants, termites, rodents and geophagic material from the SUG and GG environments. Superscript notations: (1) “T” denotes LCR for wild foods plus medicinal plants consumed with no infusion, and (2) “In” denotes wild foods consumed with medicinal plants as infusion.

The TGCs’ lifetime cancer risks (LCRs) for individual wild food types across five categories in the SUG and GG environments are presented in Figure 5. Wild foods and geophagic materials, taken together from both geological environments, exceeded the tolerable risk level of 1 × 10^−4^. Their LCR values ranged from 2.11 × 10^−1^ in *Z. mucronata* to 6.83 × 10^−7^ in geophagic material. The infusion process reduced the LCR below risk levels in all medicinal plants except for *A. thunbergii.*

The TGCs in all medicinal plants consumed as whole (total, T) significantly exceeded the safety threshold, suggesting a high potential lifetime cancer risk from long-term consumption in both environments. There were apparent differences in LCR between total (T) and infusion (In), with *E. abyssinica* showing a high total LCR (2.11 × 10^−1^) but a negligible infusion LCR (6.83 × 10^−7^). This suggested that high cancer risk is primarily associated with consuming whole plant parts rather than the liquid extract. The consumption of termites (*M. falciger*) had a much higher risk under the SUG environment (1.15 × 10^−5^) than under the GG environment (3.15 × 10^−6^), likely reflecting localized environmental contamination, while rodents (*M. natalensis*) had a consistently high LCR under both geological environments (>1.0 × 10^−3^).

**Figure 6:**
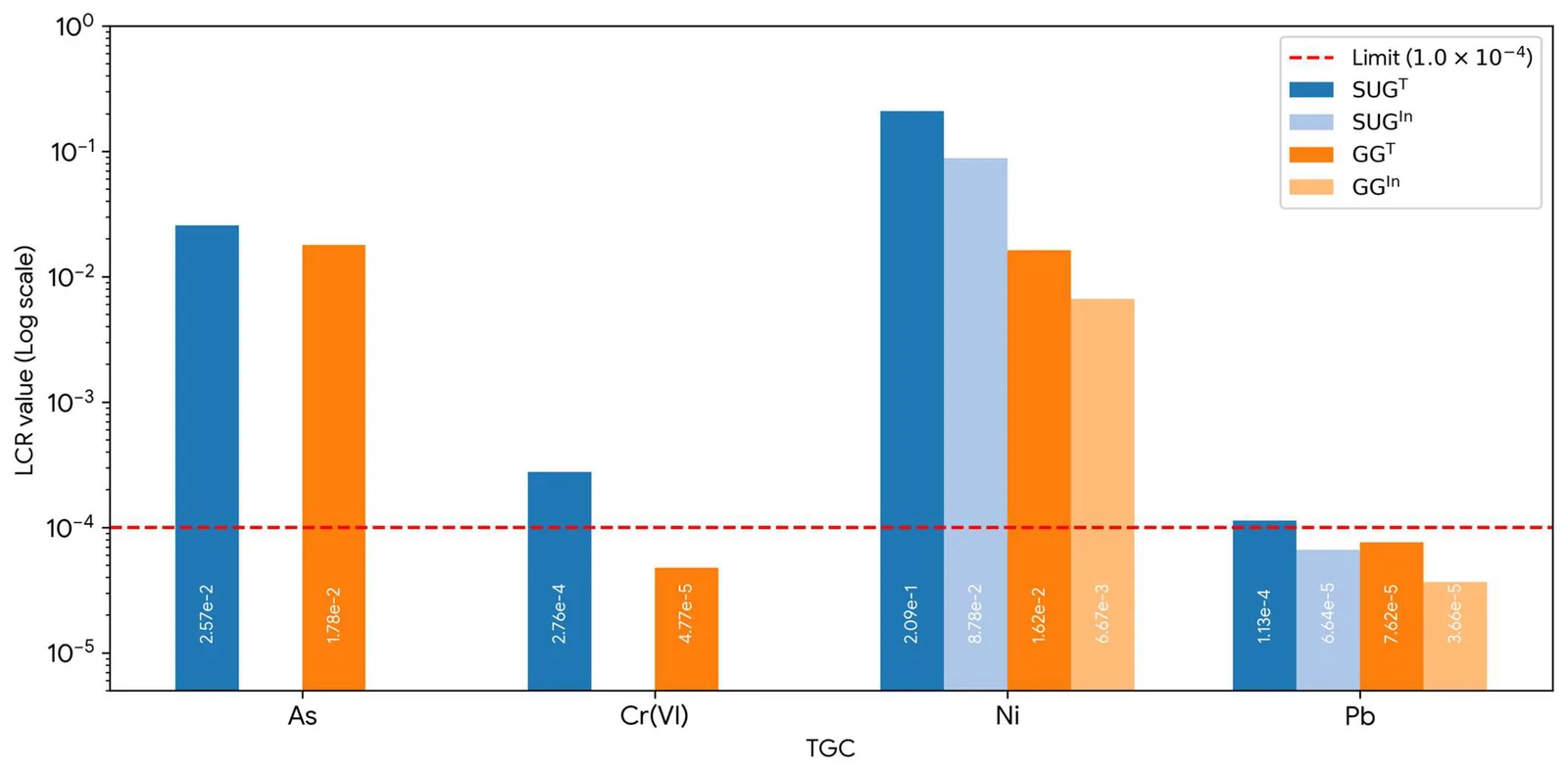
Lifetime cancer risk (LCR) for TGCs from the SUG and GG environments. Notations: (1) “Total SUG and Total GG” denotes LCR for wild foods plus medicinal plants consumed with no infusion, and (2) “Infusion SUG and Infusion GG” denotes wild foods consumed with medicinal plants as infusion.

All TGCs had a high LCR value above the tolerable risk level, except for Cu, Fe, Mn and Zn under both SUG and GG environments (Figure 6). The infusion process reduced the LCR values for TGCs, with only Pb in the GG environment decreasing to below the tolerable risk level.

## 4. Discussion

The presence of all ten TGCs in all wild foods, as well as the moderate to high bioaccumulation reported by Chigona et al. (2026), signify potential health risks associated with their consumption [19]. However, in the case of medicinal plants, results showed that the way they are consumed (whole or infusion) has a more significant impact on the level of TGCs’ intake than the geological environment. Infusions significantly decreased the intake rate of TGCs compared to whole plant part consumption. Results confirm studies such as [27,28,29] that showed significant reductions in TGCs when consumed as infusion or herbal teas. The intake of Ni and Pb were reduced after infusion, but their concentration remained above the permissible limit as also reported by [30]. The behavior of these TGCs also displayed high solubility in water following infusion, similar to what was reported in Refs. [27,31].

The consumption of medicinal plants through infusion, as compared to consumption of the whole plant part, significantly decreases the human health risk, as shown by a drop in the HI under both geological environments. Communities consuming the whole plant parts of *A. garckeana*, *A. laricinus*, *E. abyssinica* and *L. javanica* are exposed to non-cancer health risks associated with high intake of As, Co, Fe, Mn, Ni and Pb. This points to the need to carefully select the wild foods consumed, avoiding those with high concentrations of TGCs and high HI as presented in the results.

The geological environment played a critical role on the level of human health risks associated with geophagic material consumption. The consumption of termite tree wraps from the SUG environment had about 10-fold higher risk compared to consumption of the same under the GG environment. The results confirm the findings from other studies [7] and points to the need to discourage the consumption of geophagic material obtained from the SUG (Great Dyke in Zimbabwe) and related environments. This is particularly concerning for vulnerable groups like children and pregnant women, whose safety thresholds are perceived to be even lower [32,33]. The practice is known to expose women to other negative effects of geophagic material, such as parasitic infection and physiological disorders [34,35]. It is, therefore, advisable that the communities practicing geophagy in the study area be assessed for these physiological disorders.

The wild fruit *F. sycomorus*, which is a known hyperaccumulator of heavy metals, had the highest human health risks. The said wild food’s TGC concentration and resultant high HQ values across geological environments suggest a physiological attribute that enables them to absorb and bioaccumulate toxic elements, which can affect higher trophic levels of the food chain [36,37]. It is, therefore, recommended that communities avoid consuming *F. sycomorus* fruits from the SUG environment, as this was shown to pose significant human health risks at very low ingestion rates of at least two fruits per day. Instead, communities may consume *F. sycomorus* fruits from nearby areas not contaminated by TGCs.

Consumption of all wild foods and geophagic material in the current study poses lifetime cancer risk (LCR). However, for the medicinal plants, the infusion reduced the LCR values, and for *E. abyssinica* and *A. garckeana*, this reduction led the plants to reach safe levels under both geological environments, reiterating the benefits of infusion in lowering the human health risks. These findings are supported by Ref. [29], which established that the consumption of products of the infusion process reduces cancer risks to safe levels, but in that study, Cd remained at a level posing cancer risk. In our study, the problematic TGCs that remained a cancer risk were As, Cd, Co, Cr, Ni and Pb. Communities relying on these wild foods and geophagic material are therefore at greater cancer risk. This is more so now with the impact of climate change, which has increased dependency of communities on wild foods as a coping strategy [38,39]. With the commercialization of wild foods as a livelihood strategy, the health risks are not only limited to the study area but also to urban areas where there is a market for wild foods [6].

Some limitations related to the study were noted. The study covered about 15% of the Great Dyke, one out of the three agro-ecological zones, and may have missed the effects of climate on TGC availability in the other zones. The second limitation was the 10 km proximity of the GG from the SUG environment, which may have resulted in cross-contamination between the two environments, hence the noted risks in the GG environment. The adoption of a standardized preparation protocol for all medicinal plant infusions may be another limitation. While a uniform volume of 300 mL and fixed maceration parameters (time and temperature) allowed for controlled comparisons across medicinal plant species, this approach could not have fully captured the cultural variability in traditional preparation methods. In real-world settings, consumption volumes often fluctuate between 150 and 500 mL, and variations in water temperature or steeping duration can significantly alter the extraction efficiency of contaminants.

The present study utilizes total concentration, which may overestimate risk, as the wild foods, medicinal plants and geophagic material often restrict the fraction of TGCs available for intestinal absorption. Future studies should therefore focus on direct epidemiological validation through biomarkers of internal dosage to confirm if these risks translate to significant body burdens.

## 5. Conclusions

The study shows that wild foods and geophagic material pose significant health risks from TGCs for communities along the Great Dyke and surrounding areas. The high hazard quotient and hazard index values for approximately 50% of the studied wild foods, particularly *F. sycomorus* and *A. laricinus*, indicate substantial non-carcinogenic and carcinogenic risks driven by the hyperaccumulation of As, Cd, Co, Cr, Ni and Pb. Consumption of medicinal plants in infusions reduced HQ and HI substantially, to tolerable risk levels, making them safer ingestion forms in relation to non-cancer effects. In addition to the known health benefits of these wild foods, communities should apply selective consumption, reduce ingestions and avoid *F. sycomorus* and geophagic materials which have high amounts of TGCs and an extreme cancer risk factor. There is a need to increase awareness of the risks associated with the consumption of these wild foods and geophagic material. Future studies should focus on wild food processing methods that reduce the risk of TGC exposure and on clinical studies of communities along the Great Dyke and surrounding areas. Continuous monitoring and isotopic tracing to differentiate contaminant drivers, and further exploration of the synergistic effects of cumulative metal exposure, are necessary to safeguard these communities and inform local environmental health policies.

## Figures and Tables

**Table 1:** Mean TGC in wild foods from the SUG and GG environments (mean ± standard error of mean).

Wild Food Category	Scientific Name (and Part Consumed)	TGC	SUG (mg/kg)	GG (mg/kg)
Fruits	*Strychnos cocculoides* (fruit pulp)	As	0.02 ± 0.01	0.01 ± 0
		Cd	1.4 ± 0.13	2.05 ± 0.11
		Co	0.31 ± 0.07	0.24 ± 0.08
		Cr	0.84 ± 0.22	0.5 ± 0.17
		Cu	0.41 ± 0.09	0.32 ± 0.07
		Fe	9.48 ± 1.3	12.43 ± 2.99
		Mn	12.94 ± 1.74	21.05 ± 2.36
		Ni	6.73 ± 0.44	7.83 ± 0.47
		Pb	0.04 ± 0.04	ND
		Zn	2.22 ± 0.33	2.06 ± 0.33
	*Azanza garckeana* (fruit pulp)	As	0.01 ± 0	ND
		Cd	0.39 ± 0.06	0.86 ± 0.07
		Co	1.66 ± 0.53	1.8 ± 0.72
		Cr	1.46 ± 0.33	0.7 ± 0.31
		Cu	6.9 ± 1.81	10.57 ± 3.01
		Fe	99.07 ± 21.9	32.24 ± 4.04
		Mn	54.46 ± 23.36	22.01 ± 2.04
		Ni	11.04 ± 2.97	5.65 ± 0.16
		Pb	ND	0.36 ± 0.36
		Zn	14.76 ± 1.92	15.09 ± 1.86
	*Euclea natalensis* (fruit pulp)	As	ND	0.01 ± 0.01
		Cd	0.09 ± 0.07	0.14 ± 0.13
		Co	2.48 ± 0.56	1.88 ± 0.45
		Cr	2.78 ± 0.7	0.13 ± 0.1
		Cu	7.07 ± 1.57	2.28 ± 1.14
		Fe	88.9 ± 23.81	15.3 ± 3.28
		Mn	26.71 ± 11.83	136.64 ± 35.09
		Ni	7.14 ± 0.56	5.7 ± 0.46
		Pb	ND	ND
		Zn	20.63 ± 2.64	8.33 ± 1.78
	*Ziziphus mucronata* (fruit pulp)	As	ND	ND
		Cd	0.35 ± 0.02	0.41 ± 0.07
		Co	0.52 ± 0.14	0.36 ± 0.1
		Cr	2.2 ± 0.71	0.43 ± 0.14
		Cu	3.57 ± 0.41	3.93 ± 0.78
		Fe	73.23 ± 17.52	35.2 ± 6.01
		Mn	33.16 ± 12.86	25.28 ± 2.05
		Ni	8.87 ± 2.18	3.91 ± 0.32
		Pb	ND	ND
		Zn	9.19 ± 0.88	8.06 ± 1.42
	*Ficus sycomorus* (fruit pulp)	As	0.24 ± 0.01	0.13 ± 0.04
		Cd	1.32 ± 0.24	0.83 ± 0.12
		Co	20.01 ± 2.66	6.1 ± 0.62
		Cr	146.76 ± 20.68	7.77 ± 0.91
		Cu	40.87 ± 7.57	29.43 ± 4.27
		Fe	10,723.48 ± 1878.72	256.06 ± 32.42
		Mn	262.03 ± 19	199.72 ± 49.34
		Ni	133.7 ± 18.55	22.64 ± 3.06
		Pb	23.76 ± 4.27	8.78 ± 1.92
		Zn	114.25 ± 8.86	71.98 ± 11.24
	*Flacourtia indica* (fruit pulp)	As	ND	ND
		Cd	0.1 ± 0.05	0.12 ± 0.07
		Co	2.28 ± 0.96	1.22 ± 0.26
		Cr	3.83 ± 1.3	0.97 ± 0.52
		Cu	5.14 ± 2.92	2.28 ± 0.82
		Fe	75.3 ± 43.89	29.81 ± 14.25
		Mn	31.03 ± 7.47	28.46 ± 3.98
		Ni	11.18 ± 5.4	5.43 ± 0.42
		Pb	1.34 ± 0.68	0.19 ± 0.12
		Zn	25.31 ± 11.73	8.3 ± 1.3
Medicinal plants	*Erythrina abyssinica* (bark)	As	2.42 ± 0.35	1.45 ± 0.36
		Cd	2.29 ± 0.29	2 ± 0.29
		Co	13.39 ± 2.27	8.23 ± 0.98
		Cr	55.55 ± 11.62	24.89 ± 4.54
		Cu	21.63 ± 5.69	10.19 ± 1.73
		Fe	2950.91 ± 876.51	1444.3 ± 229.99
		Mn	194.43 ± 31.15	234.43 ± 50.49
		Ni	91.91 ± 23.96	23.36 ± 5.23
		Pb	25.86 ± 3.55	20.32 ± 2.26
		Zn	36.28 ± 6.13	23.02 ± 4.63
	*Azanza garckeana* (leaves)	As	0.45 ± 0.08	0.16 ± 0.05
		Cd	1.38 ± 0.04	1.33 ± 0.29
		Co	4.44 ± 0.29	6.1 ± 0.81
		Cr	5.75 ± 0.82	11.46 ± 3.46
		Cu	4.54 ± 0.48	11.2 ± 3.08
		Fe	492.05 ± 83.2	866.49 ± 564.3
		Mn	282.4 ± 25.12	92.41 ± 19.88
		Ni	9.25 ± 0.63	9.66 ± 0.96
		Pb	5.04 ± 0.91	9.52 ± 2.31
		Zn	16.66 ± 0.78	38.42 ± 10.47
	*Asparagus laricinus* (roots)	As	5.31 ± 2.53	2.47 ± 0.28
		Cd	0.87 ± 0.21	1.13 ± 0.09
		Co	9.27 ± 1.8	9.47 ± 0.83
		Cr	83.33 ± 25.29	89.74 ± 13.28
		Cu	24.04 ± 4.62	26.02 ± 4.53
		Fe	5130.53 ± 1067.51	7154.28 ± 1381.94
		Mn	134.27 ± 32.13	263.18 ± 24.23
		Ni	64.14 ± 16.56	55 ± 6.92
		Pb	11.8 ± 2.19	11.63 ± 1.16
		Zn	133.75 ± 75.4	43.86 ± 8.36
	*Amaranthus thunbergii* (leaves)	As	0.15 ± 0.03	0.36 ± 0.06
		Cd	0.66 ± 0.1	0.97 ± 0.25
		Co	3.89 ± 1.03	5.7 ± 0.97
		Cr	26.19 ± 8.56	14.38 ± 3.37
		Cu	299.52 ± 201.2	11.06 ± 2.72
		Fe	1283.55 ± 551.16	592.66 ± 90.5
		Mn	851.54 ± 264.6	842.14 ± 124.89
		Ni	42.94 ± 10.43	24.05 ± 4.24
		Pb	0.92 ± 0.59	4.03 ± 1.71
		Zn	23.36 ± 4.35	43.25 ± 5.74
	*Lippia javanica* (leaves)	As	0.51 ± 0.12	0.3 ± 0.06
		Cd	0.81 ± 0.12	0.49 ± 0.16
		Co	8.7 ± 1.55	4.34 ± 0.6
		Cr	39.01 ± 5.98	9.84 ± 2.18
		Cu	18.96 ± 3.6	18.19 ± 2.89
		Fe	2359.69 ± 509.62	827.82 ± 139.07
		Mn	265 ± 28.99	315.72 ± 43.5
		Ni	106.82 ± 28.64	20.54 ± 3.53
		Pb	2.84 ± 0.72	2.16 ± 0.73
		Zn	40.96 ± 4.77	34.35 ± 6.1
Termites	*Macrotermes falciger* (whole)	As	0.04 ± 0.02	0.27 ± 0.07
		Cd	1.8 ± 0.3	0.95 ± 0.14
		Co	12.06 ± 1.6	2.51 ± 0.58
		Cr	96.97 ± 7.27	1.86 ± 0.82
		Cu	145.06 ± 28.8	91.06 ± 30.19
		Fe	10,597.39 ± 579.53	2177.65 ± 452.59
		Mn	1522.86 ± 274.53	1320.62 ± 148.47
		Ni	106.98 ± 9.93	6.55 ± 1.01
		Pb	1.35 ± 0.59	ND
		Zn	767.42 ± 154.72	791.32 ± 86.63
Rodents	*Mastomys natalensis* (whole except offals)	As	0.05 ± 0.01	0.53 ± 0.23
		Cd	0.92 ± 0.06	1.23 ± 0.14
		Co	7.06 ± 0.67	6.8 ± 0.44
		Cr	18.75 ± 1.01	19.36 ± 2.06
		Cu	11.2 ± 2.52	10.01 ± 1.75
		Fe	1095.06 ± 109.18	1046.47 ± 154.34
		Mn	22.05 ± 1.75	42.54 ± 11.7
		Ni	14.71 ± 1.78	11.37 ± 1.36
		Pb	11.63 ± 0.87	10.89 ± 0.63
		Zn	211.09 ± 20.83	141.87 ± 16.13
Geophagy	*Termite-soil-tree-wrap* (whole)	As	0.09 ± 0.02	0.33 ± 0.08
		Cd	0.2 ± 0.03	0.07 ± 0.02
		Co	35.65 ± 6.71	3.65 ± 0.53
		Cr	104.56 ± 26.24	4.19 ± 1.21
		Cu	1.94 ± 0.26	3.98 ± 1.06
		Fe	14,329 ± 3993.6	1605.06 ± 279.61
		Mn	593.04 ± 100.95	216.83 ± 39.35
		Ni	449.27 ± 181.8	4.43 ± 0.65
		Pb	5.69 ± 1.02	6.26 ± 0.82
		Zn	6.75 ± 1.24	3.62 ± 0.85

ND—not detected (detection limit < 0.01 mg/kg); Source: modified after Chigona et al. (2026) [19].

**Table 3:** Total TGC concentration in medicinal plants infusions from the SUG and GG environments. Numbers on bars are means, and error bars denote standard error of mean.

Wild Food	TGC (Permissible Limit ^µ^, mg/kg)	SUG (mg/kg)	GG (mg/kg)	Kruskal–Wallis
*Azanza garckeana*	Cd (0.2)	ND	ND	n/a
	Co (0.01)	ND	ND	n/a
	Cr (1.0)	ND	ND	n/a
	Cu (5.0)	0.59 ± 0.06	1.45 ± 0.40	NS
	Fe (425.5)	47.16 ± 7.97	83.04 ± 54.08	*
	Mn (500)	127.08 ± 11.30	41.59 ± 8.95	*
	Ni (0.5)	ND	ND	n/a
	Pb (0.3)	2.32 ± 0.42	4.38 ± 1.06	*
	Zn (100)	12.77 ± 0.6	29.44 ± 8.02	*
*Erythrina abyssinica*	Cd (0.2)	ND	ND	n/a
	Co (0.01)	0.11 ± 0.02	ND	n/a
	Cr (1.0)	ND	ND	n/a
	Cu (5.0)	4.67 ± 1.23	8.59 ± 1.46	NS
	Fe (425.5)	7.01 ± 2.08	6.92 ± 1.10	NS
	Mn (500)	4.69 ± 0.75	23.42 ± 5.04	*
	Ni (0.5)	0.09 ± 0.02	ND	n/a
	Pb (0.3)	3.33 ± 0.46	1.22 ± 0.14	NS
	Zn (100)	1.49 ± 0.25	4.70 ± 0.94	NS
*Asparagus laricinus*	Cd (0.2)	ND	ND	n/a
	Co (0.01)	0.49 ± 0.10	0.21 ± 0.02	*
	Cr (1.0)	ND	ND	n/a
	Cu (5.0)	6.65 ± 1.28	9.17 ± 1.48	*
	Fe (425.5)	10.37 ± 2.16	28.3 ± 5.34	*
	Mn (500)	0.65 ± 0.16	14.65 ± 1.54	*
	Ni (0.5)	1.96 ± 0.51	ND	n/a
	Pb (0.3)	5.50 ± 1.02	3.17 ± 0.32	NS
	Zn (100)	8.26 ± 4.66	3.09 ± 0.58	*
*Amaranthus thunbergii*	Cd (0.2)	ND	ND	n/a
	Co (0.01)	0.20 ± 0.05	0.29 ± 0.05	*
	Cr (1.0)	ND	ND	n/a
	Cu (5.0)	51.62 ± 34.68	7.92 ± 1.95	*
	Fe (425.5)	29.19 ± 12.54	23.54 ± 3.59	*
	Mn (500)	13.92 ± 4.32	70.63 ± 10.47	NS
	Ni (0.5)	7.17 ± 1.74	5.85 ± 1.03	*
	Pb (0.3)	0.71 ± 0.46	ND	n/a
	Zn (100)	3.68 ± 0.68	11.94 ± 1.58	*
*Lippia javanica*	Cd (0.2)	ND	ND	n/a
	Co (0.01)	ND	1.01 ± 0.14	n/a
	Cr (1.0)	ND	ND	n/a
	Cu (5.0)	6.67 ± 1.27	13.57 ± 2.16	NS
	Fe (425.5)	8.43 ± 1.82	19.08 ± 3.21	*
	Mn (500)	52.94 ± 5.79	27.2 ± 3.75	*
	Ni (0.5)	ND	1.39 ± 0.24	*
	Pb (0.3)	ND	ND	*
	Zn (100)	1.36 ± 0.16	0.41 ± 0.07	*

*—significant difference (*p* < 0.05); NS—not significantly different (*p* > 0.05); ND—not detected (detection limit < 0.01 mg/kg); n/a—not applicable; µ—source [26].

**Table 4:** Wild food availability, consumption frequency and ingestion rate for SUG and GG environments.

Scientific Name	Part Consumed	Availability Calendar (Days/Year, Period)	Consumption Frequency (Items/Week)	Average Weight of Food Item (g)	Ingestion Rate (g/Week)	Ingestion Rate (g/Day)
*Strychnos cocculoides*	Pulp	153 (Jun–Oct)	2 ± 1	117.77 ± 0.71	355.54	50.79 ± 7.90
*Azanza garckeana*	Pulp	153 (Jun–Oct)	3 ± 1	14.21 ± 0.21	42.63	6.09 ± 1.35
*Euclea natalensis*	Whole	153 (Jun–Oct)	80 ± 12	3.12 ± 0.07	249.60	35.66 ± 5.15
*Ziziphus mucronata*	Pulp	153 (Jun–Oct)	8 ± 2	2.76 ± 0.07	22.32	3.19 ± 0.55
*Ficus sycomorus*	Whole	153 (Jun–Oct)	11 ± 4	34.82 ± 0.35	383.02	54.72 ± 17.42
*Flacourtia indica*	Pulp	122 (Apr–Jul)	23 ± 7	8.80 ± 0.21	202.40	28.91 ± 8.38
*Macrotermes falciger*	Whole	92 (Oct–Dec)	225 ± 34	0.03 ± 0.00	6.75	0.96 ± 0.14
*Mastomys natalensis*	Carcass	153 (Apr–Aug)	4 ± 1	34.50 ± 0.21	138.00	19.71 ± 4.79
Geophagic material	Whole	183 (Apr–Sep)	2 ± 1	80.00 ± 6.87	160.00	22.86 ± 4.07

**Table 5:** Consumption and ingestion rate for medicinal plants.

Food Item (Scientific Name)	Availability Calendar (Days/Year, Period)	Part Consumed (Form)	Amount Used in Infusion (g/L)	Ingestion Rate
*Erythrina abyssinica*	365 (Jan–Dec)	Bark (infusion)	23.78 ± 0.22	0.30 L/day
		Bark (whole)	n/a	7.14 g/day
*Asparagus laricinus*	365 (Jan–Dec)	Root (infusion)	27.96 ± 0.21	0.30 L/day
		Root (whole)	n/a	8.40 g/day
*Azanza garckeana*	365 (Jan–Dec)	Leaf (infusion)	21.86 ± 0.20	0.30 L/day
*Lippia javanica*	365 (Jan–Dec)	Leaf (infusion)	21.92 ± 0.21	0.30 L/day
*Amaranthus thunbergii*	92 (Nov–Jan)	Leaf (infusion)	12.30 ± 0.16	0.30 L/day

n/a—whole part consumed as powder in porridge as instructed by key informant.

## Data Availability

The data presented in this study are available on request from the corresponding author because it is owned by the funding organization, the Environmental Management Agency (EMA) of Zimbabwe.
